# Mito–Nuclear Interactions in Livestock: Implications for Adaptation, Productivity, and Precision Breeding

**DOI:** 10.3390/ijms27198921

**Published:** 2026-10-08

**Authors:** Manjit Panigrahi, Divya Rajawat, Amritanshu Upadhyay, K. Devadharshini, G. Ranjith, Pratik Kumar Rath, Surya Kant Verma, Sonali Sonejita Nayak

**Affiliations:** Division of Animal Genetics, Indian Veterinary Research Institute, Izatnagar, Bareilly 243122, UP, India; manjit.panigrahi@icar.org.in (M.P.);

**Keywords:** mito–nuclear interactions, mitochondrial DNA, livestock adaptation, productivity traits, genomic selection, precision breeding

## Abstract

Mitochondria are central regulators of cellular energy metabolism, stress responses, and physiological adaptation, with their functions depending on the coordinated interaction between mitochondrial DNA (mtDNA) and nuclear DNA (nDNA). These mito–nuclear interactions are essential for maintaining oxidative phosphorylation (OXPHOS), cellular homeostasis, and overall organismal fitness. Increasing evidence suggests that mito–nuclear compatibility influences a wide range of economically important traits in livestock, including growth, milk production, reproduction, feed efficiency, disease resistance, and environmental adaptation. This review provides a comprehensive overview of mitochondrial and nuclear genome organization, mechanisms of mito–nuclear communication, and their biological significance in livestock species. Key mechanisms discussed include mito–nuclear co-evolution, anterograde and retrograde signaling, reactive oxygen species (ROS)-mediated signaling, epigenetic regulation, mitophagy, mitochondrial biogenesis, and mito–nuclear incompatibility. The review further highlights the role of mito–nuclear interactions in adaptation to heat stress, high-altitude environments, metabolic challenges, and disease resistance, as well as their influence on productivity traits such as milk yield, growth performance, fertility, reproductive efficiency, and longevity. Advances in genomic and multi-omics technologies, including genome-wide association studies (GWAS), mitogenomics, transcriptomics, proteomics, metabolomics, single-cell omics, network biology, and systems genomics, have greatly enhanced our understanding of mitochondrial function and its contribution to complex traits. Furthermore, integrating mitochondrial information into genomic selection, marker-assisted breeding, climate-smart livestock improvement, and precision livestock farming (PLF) offers new opportunities to enhance productivity, resilience, and sustainability. Understanding the genetic and functional basis of mito–nuclear interactions will facilitate the development of next-generation precision breeding strategies aimed at improving livestock performance under changing environmental conditions.

## 1. Introduction

Mitochondria are essential regulators of cellular energy production, redox balance, calcium homeostasis, apoptosis, biosynthesis and responses to physiological and environmental stress. These functions are particularly important in livestock because economically important traits such as growth, milk production, reproduction, feed efficiency, disease resistance and climatic adaptation impose substantial and variable energetic demands on cells and tissues [1]. Mitochondrial performance is therefore closely connected to animal productivity, health and resilience. However, mitochondria are not genetically autonomous: their function depends on coordinated communication and cooperation between mitochondrial DNA (mtDNA) and nuclear DNA (nDNA) [2,3]. Mitochondria originated from an ancestral *α-proteobacterium* through endosymbiosis, followed by the progressive loss or transfer of most mitochondrial genes to the nuclear genome [4]. Consequently, mammalian mtDNA retains only 37 genes, including 13 protein-coding genes that form essential components of the oxidative phosphorylation (OXPHOS) system, 22 transfer RNAs, and two ribosomal RNAs [5]. In contrast, more than one thousand proteins required for mitochondrial replication, transcription, translation, energy metabolism, biogenesis and quality control are encoded by nuclear genes and subsequently imported into mitochondria [3,6]. Normal mitochondrial function therefore depends on the precise regulation, assembly and functional compatibility of products encoded by the two physically separate genomes.

OXPHOS provides a prominent example of this interdependence because its respiratory complexes contain subunits encoded by both mtDNA and nDNA. Efficient ATP production requires these subunits to interact correctly, whereas incompatible combinations may disrupt electron transport, reduce ATP synthesis, increase reactive oxygen species (ROS) production, and compromise cellular homeostasis. Because mtDNA has a comparatively high mutation rate, compensatory changes may arise in nuclear genes to preserve mitochondrial function. This reciprocal evolutionary adjustment, termed mito–nuclear co-evolution, produces coadapted mitochondrial and nuclear genetic combinations that support normal respiration, metabolism, and organismal fitness [6]. Mito–nuclear communication also extends beyond structural interactions within OXPHOS: anterograde signals from the nucleus regulate mitochondrial biogenesis and activity, whereas retrograde signals from mitochondria modify nuclear gene expression in response to energetic imbalance, oxidative stress, and cellular damage [3]. Mito–nuclear compatibility has a particular biological relevance in livestock populations exposed to artificial selection, crossbreeding, and diverse production environments. Selection for rapid growth, high milk yield or increased reproductive output intensifies metabolic demands and may expose deficiencies in mitochondrial function. Similarly, crossbreeding may combine maternally inherited mitochondrial haplotypes with nuclear genetic backgrounds with which they have not co-evolved, potentially generating mito–nuclear discordance. Evidence from African admixed cattle indicates that combinations of taurine mitochondrial genomes and zebu nuclear genomes have been influenced by natural selection, demonstrating that mito–nuclear compatibility can contribute to adaptation and physiological performance [7]. Compatibility may therefore influence whether animals can maintain energy production, fertility, immune function and tissue integrity under conditions such as heat stress, nutritional restriction, disease challenge and hypoxia. Heat stress is especially relevant because it disrupts mitochondrial activity, enhances oxidative stress, alters metabolic regulation and reduces animal performance [8]. The importance of mito–nuclear compatibility also extends to livestock improvement. Most conventional genetic and genomic evaluations model nuclear marker effects and biparental inheritance, whereas mtDNA is maternally inherited, undergoes little or no recombination, and may exhibit heteroplasmy. Consequently, mitochondrial haplotypes, maternal-line effects and interactions between mitochondrial and nuclear variants are rarely represented explicitly in estimated breeding values or genomic prediction models [9,10]. Incorporating this information could improve the identification of animals possessing favorable combinations of energy efficiency, production potential and environmental resilience. It may also support the design of reciprocal crosses, conservation of valuable maternal lineages, selection of climate-resilient animals and development of genomic prediction models that account for both mitochondrial and nuclear sources of variation. Despite growing interest, current knowledge of mito–nuclear interactions in livestock remains fragmented. Most studies have evaluated mitochondrial haplotypes, mtDNA variants, mitochondrial copy number, or nuclear loci associated with mitochondrial traits separately. At the same time, relatively few have directly quantified mtDNA × nDNA interactions in large, well-phenotyped populations. Available reference populations are generally small, mitochondrial diversity in indigenous breeds remains inadequately characterized, and functional validation of associated variants is limited. Tissue-specific heteroplasmy, developmental changes and interactions with nutrition, climate and management further complicate the interpretation of mitochondrial effects. In addition, the lack of standardized mtDNA reference panels, analytical pipelines and integrated genomic–transcriptomic–proteomic–metabolomic datasets restricts the translation of mito–nuclear research into routine livestock breeding [11,12]. These gaps explain why mitochondrial information has not yet been widely incorporated into genomic selection and precision-breeding programs. Although there have been significant advances, several research gaps remain in our understanding of mito–nuclear interactions in livestock. Most evidence comes from model organisms, with little functional validation in livestock species. Mitochondrial genomic information used in genomic selection and precision breeding is still under-explored. Further, the molecular basis of mito–nuclear incompatibility, climate resilience, and economically important traits needs to be thoroughly investigated by integrating multi-omics, single-cell technologies, and artificial intelligence. Addressing these gaps will allow for the development of more accurate, climate-resilient, and sustainable livestock breeding strategies. Accordingly, this review aimed to critically synthesize current knowledge of mito–nuclear interactions and their significance in livestock. Its specific objectives were to: (i) describe the organization and functional interdependence of the mitochondrial and nuclear genomes; (ii) explain the principal mechanisms of mito–nuclear communication, co-evolution and incompatibility; (iii) evaluate evidence linking mito–nuclear variation with adaptation, productivity, reproduction, health and disease resistance; (iv) examine the genomic and multi-omics approaches available for investigating these interactions; (v) assess opportunities for incorporating mitochondrial information into genomic selection, crossbreeding, climate-smart improvement and precision livestock breeding; and (vi) identify the major biological, analytical and implementation challenges that must be addressed to translate mito–nuclear research into practical livestock-improvement strategies.

## 2. Overview of Mitochondrial and Nuclear Genomes

### 2.1. Structure and Function of mtDNA

mtDNA is a semiautonomous, compact circular genome encoding essential components of the OXPHOS machinery. Traces of its origin have been explained by the endosymbiotic theory, indicating that mitochondria have evolved specifically from ancestral *alphaproteobacteria* [13] and retain their own genome [14]. Animals have evolved a compact mitochondrial genome between 11 and 55 kb in length [13]. The noncoding control region in the mitochondrial genome, commonly known as the D-loop, serves as the primary regulatory site for mtDNA replication and transcription [15]. As a consequence of the absence of protective histones coupled with limited DNA repair mechanisms, the mtDNA exhibits higher mutation rates than nDNA [16]. The versatile functionality of mitochondria has made this organelle essential, as it plays a central role in aerobic respiration and cellular ATP generation, and its genes encode core subunits of OXPHOS machinery [17]. In addition, mitochondria play a crucial role in calcium homeostasis, programmed cell death (apoptosis), biosynthesis of various lipids and iron–sulfur clusters, and cellular responses to stressors [18].

### 2.2. Maternal Inheritance of Mitochondria

Maternal inheritance is a defining feature of mitochondrial genetics, whereby mtDNA is transmitted almost exclusively through the maternal lineage. This happens because the mitochondria introduced by the sperm are generally degraded or eliminated shortly after fertilization due to ubiquitination and autophagy [19,20]. The genes *PRKN* and *MUL1*, encoding E3 ubiquitin ligases, play key roles in maintaining this inheritance by triggering the autophagic elimination of paternal mitochondria shortly after fertilization [21]. Evolutionary theory suggests that because of this method of inheritance, harmful mutations that specifically impair male fertility can build up over generations without being effectively weeded out by natural selection [22]. Additionally, there are indications that maternal inheritance of mtDNA profoundly influences the transmission patterns of metabolic and neuromuscular disorders [23]. Although mitochondrial inheritance is predominantly maternal, recent evidence suggests that rare cases of paternal mtDNA transmission may occur [24].

### 2.3. Heteroplasmy and Genetic Variations in mtDNA

The coexistence of more than one variant of the mtDNA within the same cell, tissue, or individual is known as heteroplasmy. Different cells, tissues, or individuals show different heteroplasmy levels because different tissues differ in different rates of mitochondrial turnover and energy requirements [25,26]. Heteroplasmy mainly develops due to novel mutations, replication errors, and the mitochondrial genetic bottleneck effect during maternal genome transmission from the paternal origin [19,20]. This transmission leads to various mutant loads among offspring and tissues. The impact of mutant mtDNA is reflected in the individual’s phenotype when the proportion of mutation in mtDNA crosses the threshold, especially in energy-demanding tissues. In livestock, heteroplasmy is associated with regulation of metabolic efficiency, fertility, adaptation, and disease susceptibility [9,27,28,29,30]. Therefore, the variable nature of mitochondrial genome inheritance, tissue or cell specificity, and the difficulties in distinguishing low-frequency variants from sequencing errors pose significant challenges for genomic evaluation and mitochondria-based breeding.

### 2.4. OXPHOS System

The primary role of mitochondria is OXPHOS. In this process, electrons released from the breakdown of nutrients are passed along the protein complexes of the electron transport chain, driving the production of ATP, the cell’s primary energy currency [31]. mtDNA encodes several critical subunits of the OXPHOS machinery, including key components of electron transport chain complexes I, III, IV, and V [5]. The mitochondrial genes *ND1*, *ND2*, *ND3*, *ND4*, *ND4L*, *ND5*, and *ND6* encode subunits of Complex I (NADH dehydrogenase) [32]. The mitochondrial gene CYTB (*MT-CYB*) codes for cytochrome b, a vital catalytic subunit of Complex III that plays a central role in electron transport [33]. The mitochondrial genes ATP6 (*MT-ATP6*) and ATP8 (*MT-ATP8*) code for specific subunits of ATP synthase (Complex V) [34]. In domestic animals, mtDNA encodes proteins that are indispensable for OXPHOS [15]. Any impairment in OXPHOS is linked to a wide array of mitochondrial diseases in mammals, as ATP production depends heavily on the proper functioning of the mitochondrial genome [35].

### 2.5. Coordination Between mtDNA and nDNA

Mito–nuclear interactions refer to the intricate functional and evolutionary coordination of mtDNA and nDNA that is required for mitochondrial structure, bioenergetics, and cellular metabolism [36]. Proper mito–nuclear compatibility is crucial for efficient mitochondrial gene expression, correct assembly of the respiratory chain complexes, and maintenance of cellular energy homeostasis [37]. Such interactions influence many fundamental biological processes such as aging, fertility, metabolism, adaptation to the environment, and susceptibility to mitochondrial diseases [38]. mtDNA accumulates mutations rapidly, and nuclear genes that interact with the mitochondrial genome often evolve rapidly to compensate for deleterious changes in mtDNA, helping to restore functional harmony [39]. Mito–nuclear mismatch alters the expression of the nuclear genes *GPX3*, *HSPB6*, *ISG15*, and *IFI6*, suggesting they play a role in stress-response pathways associated with mitochondrial dysfunction [40].

## 3. Mechanisms of Mito–Nuclear Interactions

**Figure 1:**
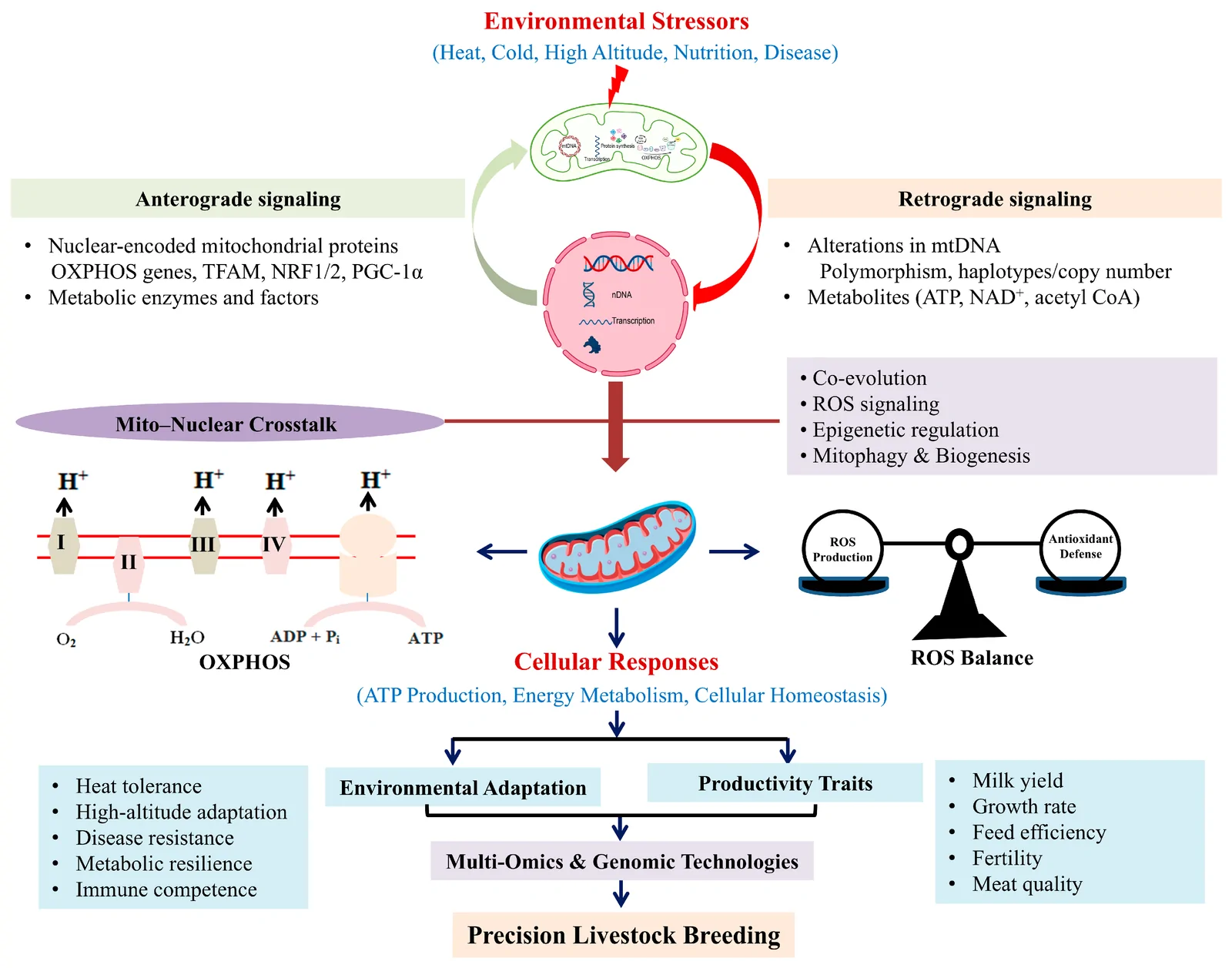
Schematic representation of mitochondrial–nuclear crosstalk, illustrating mechanisms linking environmental stressors to cellular responses, and livestock applications.

Mito–nuclear interactions represent a highly coordinated communication system between the mitochondrial and nuclear genomes that is essential for maintaining cellular homeostasis, energy metabolism, stress adaptation, and organismal survival [3]. Mitochondrial function depends on precise coordination between the two genomes through multiple molecular mechanisms, including co-evolution, intracellular signaling, epigenetic regulation, mitochondrial quality control, and mito–nuclear compatibility. Figure 1 illustrates the mitochondrial–nuclear communication pathways, linking environmental stressors with mitochondrial function, cellular responses, adaptations, and the application of genomic and multi-omics technologies in precision livestock breeding. Table 1 summarizes key studies highlighting the role of mitochondrial and mito–nuclear interactions in livestock adaptations and their practical implications.

### 3.1. Co-Evolution of Mitochondrial and Nuclear Genomes

One of the most fundamental aspects of mito–nuclear interaction is the co-evolution of mitochondrial and nuclear genomes. Despite Muller’s ratchet, mitochondrial function is maintained through compensatory coevolution, where nDNA evolves to counteract deleterious mitochondrial mutations [39,51,52]. Mitochondrial origin by endosymbiosis led to the transfer of most mitochondrial genes to the nucleus, resulting in OXPHOS complexes composed of subunits encoded by both mtDNA and nDNA. Although mitochondria possess their own genome, the nuclear genome encodes more than 1000 mitochondrial proteins [53]. This functional interdependence necessitates compensatory changes in nDNA in response to rapid mitochondrial mutations and the coordinated co-evolution of both genomes to ensure efficient energy production. This mito–nuclear co-evolution plays an important role in species adaptation to different environmental stressors such as temperature, hypoxia, and nutritional availability [6,38]. While most of the current knowledge has been acquired from model organisms, recent studies on livestock have provided additional evidence of how mito–nuclear co-evolution contributes to adaptation in domesticated animals. For instance, genome-wide local ancestry analyses have revealed that nuclear genes involved in mitochondrial activity of African admixed cattle have a considerably higher level of taurine ancestry, even though there is considerable nuclear zebu introgression, suggesting that selection pressure favors compatible mito–nuclear gene combinations to maintain high mitochondrial function and adaptation [54]. Furthermore, mito–nuclear incompatibility has been proposed as a key factor underlying the retention of taurine mitochondrial haplotypes in African admixed cattle [55]. Studies in different organisms have shown that disruption of this compatibility can impair OXPHOS, increase ROS, reduce fitness, and contribute to mitochondrial diseases, emphasizing coordinated evolution between mtDNA and nDNA [56,57].

### 3.2. Anterograde and Retrograde Signaling

Communication between mitochondria and the nucleus is bidirectional and occurs through two pathways: anterograde and retrograde signaling. Anterograde signaling refers to the flow of information from the nucleus to mitochondria, by which the nuclear genome regulates mitochondrial structure, biogenesis, and function [3]. Nuclear-encoded proteins involved in mitochondrial replication, transcription, translation, and OXPHOS assembly are synthesized in the cytosol and transported into mitochondria [58,59]. Regulatory molecules such as nuclear respiratory factors (*NRF1* and *NRF2*) and peroxisome proliferator-activated receptor gamma coactivator 1-alpha (*PGC-1α*) coordinate mitochondrial biogenesis and energy metabolism in response to cellular demands [60,61]. PGC-1α is an important regulator of mitochondrial biogenesis that co-activates transcription factors like *NRF1*, *NRF2*, and ERRα. These molecules thereby enhance the expression of genes related to mitochondrial function and biogenesis. In poultry, PGC-1α has been associated with the regulation of skeletal muscle development, muscle fiber characteristics, mitochondrial function, and meat quality traits, indicating the importance of nuclear regulation of mitochondrial biogenesis in livestock productivity [62]. On the other hand, the communication from mitochondria to the nucleus is known as retrograde signaling, through which cells activate stress response pathways, metabolic reprogramming, inflammatory responses, and apoptotic mechanisms to restore homeostasis [63]. Mitochondrial dysfunction caused by oxidative stress, ATP depletion, calcium imbalance, or membrane depolarization generates intracellular signals that alter nuclear gene expression [64,65]. Any defects in retrograde signaling can lead to neurodegenerative disorders, metabolic diseases, and cancer [66]. This precise, coordinated communication ensures cellular adaptation to changing physiological and environmental conditions.

### 3.3. ROS Signaling

ROS are another important mediator of mito–nuclear communication and are produced as a byproduct of OXPHOS from ETC complexes I and III [67]. Common ROS molecules include superoxide (O_2_^−^), hydrogen peroxide (H_2_O_2_), and hydroxyl radicals (·OH). Excessive ROS can induce oxidative damage to proteins, lipids, and nucleic acids, which leads to mitochondrial dysfunction, apoptosis, aging, and various degenerative diseases [57,68,69]. Later studies revealed that, at moderate levels, ROS function as key signaling molecules regulating many cellular activities such as proliferation, immune responses, hypoxic adaptation, and mitochondrial biogenesis. They also influence nuclear gene expression by activating signaling cascades such as mitogen-activated protein kinase (*MAPK*), nuclear factor kappa B (*NF-κB*), and hypoxia inducible factor 1α (*HIF-1α*) [65,70]. Similarly, in goat Sertoli cells, heat stress induced ROS accumulation that disrupted mitochondrial function and decreased ATP synthesis and mitochondrial membrane potential; thereby inducing mitochondria-dependent apoptosis. Melatonin treatment alleviated these effects by reducing ROS levels and restoring mitochondrial homeostasis [71].

### 3.4. Epigenetic Regulation

Epigenetics can be defined as heritable changes in the genome resulting in altered gene expression patterns without affecting the DNA sequence [72]. These epigenetic modifications primarily occur through DNA methylation, histone modifications, and noncoding RNAs [73,74]. Smiraglia et al. (2008) demonstrated that changes in mtDNA copy number can alter the DNA methylation patterns in the nuclear genome, providing direct evidence of mitochondrial regulation of nuclear gene expression [75]. Likewise, in sheep, Wang et al. (2024) [76] identified *DNMT1* and *PPARGC1A* (PGC-1α) as direct target genes of miR-148a in ovine mammary epithelial cells using a dual-luciferase reporter assay. They also observed a negative correlation between miR-148a and the expression of both genes [76]. Several studies suggest that mitochondrial metabolic activity can modify chromatin structure and regulate gene expression patterns, which in turn regulates mitochondrial function through nDNA-encoded mitochondrial proteins [63]. Mitochondrial metabolites such as acetyl-CoA, NAD^+^, α-ketoglutarate, and S-adenosylmethionine (SAM) act as substrates and cofactors for epigenetic enzymes [64,69]. Thus, mitochondria serve not only as energy producers but also as regulators of epigenetic mechanisms. Moreover, these mitochondrial metabolites can control histone modifications and DNA methylation, possibly through epigenetic inheritance. This type of regulation in mitochondrial functions can be driven by epigenetic changes mediated by adaptive responses linked to development and gene expression patterns across generations. Therefore, understanding mito-epigenetic interactions may improve the prediction of specific traits in animal breeding that are inherited from the mother and responsive to the environment [63,69,75].

### 3.5. Mitophagy and Mitochondrial Biogenesis

Mitochondrial quality and quantity within the cell are regulated by two opposing processes of mitophagy and mitochondrial biogenesis in a precisely coordinated manner in response to the cell’s energy demands and other intracellular factors [77]. Mitophagy selectively eliminates dysfunctional mitochondria to maintain mitochondrial quality and cellular homeostasis and occurs through two pathways: ubiquitin-dependent and ubiquitin-independent [78,79]. In the ubiquitin-dependent pathway, due to impaired membrane potential, the entry of phosphatase and tensin homolog (PTEN) induced kinase 1 (*PINK1*) to the inner mitochondrial membrane is blocked, resulting in accumulation of *PINK1* in the outer membrane [80,81,82]. This initiates the mitophagy cascade through parkin recruitment, which causes ubiquitination of mitochondrial proteins for autophagic degradation [83]. Similarly, in bovine mammary epithelial cells, activation of *PINK1*-mediated mitophagy protected the cells from lipopolysaccharide-induced mitochondrial and inflammatory damage [84]. The latter, ubiquitin-independent mitophagy is mediated by specific mitochondrial outer membrane protein receptors containing microtubule-associated protein 1 (MAP) light chain 3 LC3-interacting regions (LIR) that directly bind to LC3 proteins on autophagosomes [85,86]. To compensate for mitophagy, cells simultaneously activate biogenesis pathways. Mitochondrial biogenesis forms new mitochondria from pre-existing ones through the coordinated expression of nDNA and mtDNA in response to developmental and environmental stressors that alter metabolism and energy demands [87]. This process is primarily regulated by PGC-1α, a master regulator that interacts with transcription factors such as *NRF1/2*, estrogen-related receptor alpha (ERRα), and PPARα to activate mitochondrial transcription factor A (*TFAM*), which in turn controls mtDNA replication and transcription [60,61,88]. The balance between mitophagy and mitochondrial biogenesis is critical for maintaining mitochondrial homeostasis and cellular survival under stress conditions.

### 3.6. Mito–Nuclear Incompatibility

Mito–nuclear incompatibility occurs when mitochondrial and nuclear genomes fail to work in harmony with each other due to mutations, hybridization, or evolutionary divergence. In many experimental studies, this incompatibility has been linked to altered mtDNA copy number, accelerated aging, metabolic disorders, neurodegeneration, cancer, infertility, developmental abnormalities, and reduced fitness [6,40,57,77,89]. For instance, Lee et al. (2008) have demonstrated that mito–nuclear incompatibility can directly cause hybrid sterility in yeast, emphasizing its role in speciation and evolutionary biology by contributing to reproductive isolation between divergent populations [90]. Similar studies in mice revealed that compromised mitochondrial functions lead to interspecies reproductive barriers [91]. Likewise, taurine mitochondrial haplotypes have been retained in African admixed cattle, despite extensive zebu nuclear introgression, indicating that selection favors mito–nuclear gene combinations that maintain mitochondrial function [54,55]. Moran et al. (2024) identified a lethal mito–nuclear incompatibility involving complex I of natural hybrids, highlighting the role of mito–nuclear coevolution in hybrid fitness and speciation [92]. Furthermore, understanding mito–nuclear compatibility is becoming more important in mitochondrial replacement therapy and personalized medicine [91,93].

## 4. Role in Livestock Adaptation

Understanding the evolutionary history and genetic diversity of livestock species is fundamental for exploring mito–nuclear interactions and their influence on adaptation, productivity, and precision breeding. In this context, mtDNA has emerged as a reliable molecular marker for assessing genetic variation in animals. Its highly conserved gene content, minimal gene duplications, absence of introns, and short intergenic regions make mtDNA particularly suitable for studies on molecular diversity, phylogeny, evolutionary relationships, and population genetics [94].

### 4.1. Heat and High-Altitude Adaptation

In recent decades, global temperature and humidity levels have increased substantially. These changes are closely associated with an elevated risk of heat-related disorders and reduced physical performance, thereby highlighting the need to investigate physiological responses to heat stress [41]. Repeated heat stress induces mitochondrial adaptations, as demonstrated by increased expression of markers associated with mitochondrial biogenesis, mitochondrial proteins, OXPHOS machinery, and enhanced mitochondrial bioenergetic function. Mitochondrial biogenesis is a physiological process involving the formation and expansion of the mitochondrial network within the cell cytoplasm through the growth and division of pre-existing mitochondria [95]. Mitochondria are essential organelles present in most cells and play a vital role in maintaining cellular and physiological health by regulating energy metabolism, oxidative stress, and calcium homeostasis, all of which are critical for normal body functions. The efficiency and abundance of mitochondria within cells are important for several biological processes, including growth, reproduction, and adaptation to environmental stressors [1]. Heat stress has been reported to reduce mitochondrial number while increasing mitochondrial size in the mammary tissue of heat-stressed (HS) cows, along with alterations in the expression of stress-related genes [27]. Evidence from a wide range of organisms suggests convergent evolution of mitochondrial functions associated with physiological responses to oxygen deprivation and temperature stress, including adaptations to high-altitude environments. Although the mitochondrial genome is relatively small, it encodes essential core subunits of the OXPHOS respiratory chain.

However, most mitochondrial proteins are encoded by nuclear genes and subsequently transported into mitochondria. In animals, interactions between mitochondrial and nuclear gene products, known as mito–nuclear interactions, play a critical role in fitness, adaptation, evolution, and ecological responses. Notably, the OXPHOS complexes are formed through the coordinated interaction of 13 mitochondrial-encoded and approximately 80 nuclear-encoded protein subunits in most animals [96]. Mismatched mito–nuclear interactions (gene–gene interactions) can have significant biological consequences, including reduced metabolic efficiency, impaired fecundity, and shortened lifespan. Furthermore, when different mito–nuclear combinations are evaluated under varying environmental conditions such as diet, temperature, or hypoxia, their effects are often context-dependent, reflecting complex gene–gene–environment interactions [43,44]. One of the most compelling examples of the role of mitochondrial evolution in local adaptation is observed in the bar-headed goose (*Anser indicus*), a species adapted to high-altitude environments. Researchers investigated the genetic basis of this adaptation by comparing mtDNA sequences and nuclear-encoded mitochondrial genes associated with complex IV subunits between low- and high-altitude populations. Their analysis identified several non-synonymous substitutions in the bar-headed goose, including a unique W116R substitution in the *COX3* gene, along with subtle differences in complex IV expression levels that may contribute to enhanced hypoxia tolerance and high-altitude adaptation [42]. Although heat stress primarily induces mitochondrial remodeling through enhanced biogenesis and OXPHOS, high-altitude adaptation involves evolutionary changes in OXPHOS-related genes that optimize oxygen utilization. These findings suggest that efficient mito–nuclear communication is a conserved mechanism supporting environmental resilience across species. Despite this, such studies in livestock remain limited. Future studies on mitochondrial genes and OXPHOS-related nuclear genes are needed to identify mito–nuclear variants associated with adaptation to hypoxia.

### 4.2. Disease Resistance and Immunity

Alterations in mitochondrial homeostasis have been strongly associated with neuronal cell death and the pathogenesis of neurodegenerative disorders. These alterations involve multiple aspects of mitochondrial function, including calcium regulation, dysfunction of OXPHOS, and impairment of mitochondrial quality control at both molecular and organellar levels, as well as metabolic and signaling disturbances. Furthermore, genes linked to neurodegenerative diseases have been identified through GWASs. These findings suggest that disruption of mitochondrial–nuclear interactions may play a crucial role in the etiopathology of neurodegenerative diseases [28]. Both mitochondria and the gut microbiome play essential roles in maintaining animal health, regulating metabolism, and influencing therapeutic responses. Although modulation of the gut microbiota offers promising therapeutic opportunities, improper management may also pose potential risks. Additionally, mitochondrial metabolites involved in the tricarboxylic acid (TCA) cycle, such as succinate and lactate, contribute significantly to immune regulation, thermogenesis, and disease pathophysiology [45]. A notable example in livestock is the identification of pathogenic mtDNA mutations in cattle that are similar to those associated with Leber’s Hereditary Optic Neuropathy (LHON) in humans. Among these, the T10663C mutation in the *ND4L* gene was reported in a Cika cow exhibiting exophthalmia, suggesting possible functional effects on vision and potentially other physiological systems [46]. Another well-recognized mitochondria-associated disorder in livestock is atypical neuropathy in horses. Studies have shown that this condition is primarily linked to mitochondrial dysfunction caused by the ingestion of hypoglycin-A-containing feed, leading to the development of neuropathic syndromes [47]. In veterinary studies, oxidative stress and methionine-deficient diets have been shown to induce liver swelling, alter oxygen consumption, and trigger inflammatory responses in hens. Similarly, high-cholesterol diets in pigs can lead to metabolic syndrome through mitochondrial dysfunction and consequent dysregulation of autophagy [48]. Taken together, these studies demonstrate that mitochondrial dysfunction and disrupted mito–nuclear communication result in diseases through multiple mechanisms. Although the reported disorders differ among species, the underlying mitochondrial pathways are remarkably conserved, highlighting their importance as potential biomarkers for animal health and disease susceptibility.

## 5. Mito–Nuclear Interactions and Productivity Traits

Mitochondrial and mito–nuclear interactions play important roles in regulating productivity traits, particularly milk yield and milk composition in dairy cattle. A study conducted on Holstein cattle in Croatia evaluated the influence of the mitochondrial genome (mitogenome) on milk production traits using next-generation sequencing (NGS) data from 109 cows linked to 7115 milk production records of 3006 cows across the first five lactations. The study revealed that the mitogenome contributed substantially to phenotypic variation in milk, fat, and protein yield, with estimates ranging from 5% to 7% across four analytical models (CYTO, HAPLO, AMINO, and SNPmt), despite the relatively small size of the mitochondrial genome [10]. In addition, peripheral blood mononuclear cell mitochondrial enzyme activity is significantly associated with parity and lactation performance in early-lactation Holstein dairy cows, further emphasizing the importance of mitochondrial function in dairy productivity traits [49].

Mitochondrial function and mito–nuclear interactions are also closely associated with growth performance. As mitochondria are central to cellular energy metabolism, variations in mitochondrial efficiency can influence muscle development, feed efficiency, growth rate, and fat deposition. Alterations in mitochondrial activity may affect muscle fiber composition, oxidative capacity, and post-mortem biochemical processes, thereby influencing important meat quality parameters such as tenderness, marbling, water-holding capacity, and color stability. Mitochondrial and mito–nuclear interactions play crucial roles in regulating growth and developmental traits in animals through their involvement in cellular energy metabolism and redox homeostasis. Mitochondria generate important redox regulators such as NAD^+^, NADH, NADP, NADPH, GSH, and GSSG, which control metabolic flux, biosynthetic pathways, antioxidant defense, and cellular homeostasis. Efficient coordination between mitochondrial and nuclear genomes is essential for muscle growth, feed efficiency, tissue development, and overall physiological performance [29]. In addition, emerging evidence suggests that mito–nuclear interactions may also influence aging and disease susceptibility in livestock. Bouzeraa et al. (2025) reported accelerated epigenetic aging in disease-susceptible cows, indicating a potential association between health-related stress and altered DNA methylation dynamics [50]. The study further identified age-associated promoter methylation changes, particularly in the *MAB21L1* gene, which may contribute to molecular mechanisms underlying aging processes in cattle [50].

## 6. Genomic and Multi-Omics Approaches

### 6.1. Genomics: Identifying Genetic Variation

Genomics provides the foundation for understanding the genetic architecture of economically important traits by identifying DNA sequence variations associated with phenotypic diversity. Molecular markers have revolutionized livestock genetics by enabling the characterization of genetic diversity, population structure, and trait-associated loci. Among the various DNA markers developed over the past decades, microsatellites and single nucleotide polymorphisms (SNPs) have been the most extensively utilized. Microsatellites were widely adopted for livestock genotyping due to their high polymorphism and codominant inheritance. However, the advent of high-throughput sequencing technologies and SNP arrays has shifted genomic research toward SNP-based approaches because of their greater genome-wide coverage, higher reproducibility, automation, and suitability for large-scale genomic analyses [97,98]. Advances in next-generation sequencing (NGS) and high-density SNP genotyping platforms have enabled comprehensive genome-wide investigations of complex traits. Modern genomic analyses integrate several complementary approaches, including genome-wide association studies (GWASs), selection signature analyses, and candidate gene evaluation, to identify genomic regions underlying economically important phenotypes [99]. GWASs identify statistical associations between genetic variants and phenotypic traits across populations, thereby facilitating the discovery of quantitative trait loci (QTLs) and candidate genes influencing production, reproduction, adaptation, and disease resistance. In contrast, selection signature analyses detect genomic regions that have experienced positive or artificial selection during population evolution, providing insights into domestication, environmental adaptation, and long-term breeding history.

Beyond the nuclear genome, mitogenomics has emerged as an important component of genomic research. It involves the comprehensive analysis of complete mitochondrial genomes to investigate maternal inheritance, evolutionary relationships, phylogeography, and mitochondrial genetic diversity. Owing to its maternal inheritance, compact genome size, relatively high mutation rate, and absence of recombination, mtDNA serves as an informative molecular marker for tracing maternal lineages and studying evolutionary processes. Recent advances in whole-genome sequencing have facilitated routine reconstruction of complete mitochondrial genomes, expanding their application in livestock biodiversity assessment, population genetics, and breeding programs.

### 6.2. Transcriptomics: Understanding Gene Function

Candidate loci identified through GWASs can be functionally validated using transcriptomic analyses to determine whether associated genes are actively expressed in relevant tissues and physiological conditions. Organelle genes generally exhibit higher expression levels than nuclear genes, resulting in a substantial proportion of raw reads from eukaryotic RNA-Seq experiments being derived from organelles [100]. Since organelle genomes are extensively transcribed as polycistronic RNAs, transcriptomic data can often be used to recover nearly complete mitochondrial genomes [101]. However, the use of RNA-Seq data also has some limitations associated with DNA-based approaches. For example, although the likelihood of nuclear mitochondrial DNA segments (NUMTs) or mtDNA fragments transferred to the nuclear genome being represented in nuclear transcripts is relatively low, evidence suggests that nuclear mitochondrial pseudogenes can still be detected in transcriptomic datasets [102]. Despite its potential, transcriptome data are not yet routinely exploited for mitochondrial genome assembly. A limited number of studies have reported successful reconstruction of mitogenomes either by mapping reads to conspecific reference sequences followed by assembly, or by identifying mitochondrial contigs from de novo assembled transcriptomes. However, many of these studies did not generate completely new mitochondrial genomes, and most were unable to recover certain regions, such as transfer RNA (tRNA) genes and the control region, due to insufficient read coverage. This limitation is particularly important because complete assembly of these regions is essential for obtaining full mitogenomes and highlights potential challenges associated with using RNA-Seq data in mitogenomic studies [103].

### 6.3. Proteomics and Metabolomics: Linking Molecular Function to Phenotype

Changes in gene expression ultimately influence protein abundance and metabolic pathways. Therefore, integrating transcriptomics with proteomics and metabolomics enables the identification of functional biomarkers associated with production traits, feed efficiency, reproduction, disease resistance, and mitochondrial function. While transcriptomics reveals changes in gene expression, proteomics and metabolomics provide functional insights into how these molecular changes translated into cellular physiology and ultimately influence phenotypic variation. Proteomics characterizes the complete protein complement of mitochondria, enabling the identification of proteins involved in energy metabolism, OXPHOS, stress responses, and cellular adaptation. Because mitochondria contain approximately 1000–1500 proteins encoded by both the nuclear and mitochondrial genomes, mitochondrial proteomics serves as a functional bridge between genomic variation and biological processes [104]. In livestock, comparative proteomic studies have identified biomarkers associated with feed efficiency, fertility, meat quality, disease resistance, and environmental adaptation. For example, differential expression of mitochondrial proteins involved in OXPHOS, fatty acid metabolism, and hypoxia tolerance has been reported in cattle, yak, and dzo, highlighting their contribution to metabolic efficiency and adaptation to diverse production environments [105,106]. However, the functional significance of most of these proteins remains unvalidated, making it difficult to distinguish which proteins contribute to adaptation to hypoxia. Complementing proteomics, metabolomics captures the downstream metabolic consequences of genomic, transcriptomic, and proteomic regulation by profiling small-molecule metabolites, which represent the final products of cellular metabolism. Since metabolites are highly responsive to both genetic and environmental influences, metabolomics provides a sensitive representation of the functional phenotype or metabotype [107,108]. In livestock, metabolomic profiling has facilitated the identification of biomarkers associated with transition diseases, feed efficiency, carcass quality, reproductive performance, and metabolic health. For instance, long-chain acylcarnitines and biogenic amines have been identified as predictive biomarkers of productive lifespan in dairy cattle, while metabolomic analyses have been successfully applied to evaluate residual feed intake (RFI) and other economically important production traits [109,110].

### 6.4. Single-Cell Omics and Network Biology: Deciphering Cellular Heterogeneity and Regulatory Networks

Single-cell omics has revolutionized the study of mitochondrial biology by enabling the analysis of molecular variation at single-cell resolution. Unlike conventional bulk sequencing, which averages signals across heterogeneous cell populations, single-cell approaches reveal cell-specific mitochondrial mutations, heteroplasmy, and lineage dynamics. Owing to its high mutation rate and maternal inheritance, mtDNA serves as an effective molecular barcode for tracing cellular lineages and reconstructing clonal evolution. Recent advances, including rolling circle amplification (RCA) and scMito-seq, have substantially improved mitochondrial genome coverage and the detection of mtDNA variants, although challenges such as amplification bias and incomplete genome recovery remain. While single-cell omics has been widely applied in human and model organism research, its application in livestock species is still in its infancy, offering significant opportunities for understanding cellular heterogeneity underlying production, reproduction, adaptation, and disease resistance [12,111]. The high-resolution molecular data generated through single-cell omics can be further integrated using network biology, which combines genomic, transcriptomic, proteomic, and metabolomic information into interconnected regulatory networks. Rather than examining individual genes in isolation, network-based analyses identify co-expressed genes, interacting proteins, metabolic pathways, and key regulatory molecules that collectively govern complex biological traits. In livestock, network analyses integrating transcriptomic data have revealed coordinated gene expression patterns associated with feed efficiency, mitochondrial function, and environmental adaptation. For example, network analysis of cattle muscle transcriptomes identified highly connected transcription factors, including PPARA, that regulate mitochondrial energy metabolism and OXPHOS, providing insights that may be overlooked by conventional differential expression analyses [112]. Together, single-cell omics and network biology provide complementary approaches for systems-level analysis of complex traits. While single-cell technologies resolve molecular heterogeneity at the cellular level, network biology integrates these multidimensional datasets to identify key regulatory pathways and functional interactions. The ultimate objective of multi-omics integration is to establish a systems biology framework that combines genomic variation, transcriptional regulation, protein function, metabolic pathways, and phenotypic performance. Integration of these datasets through artificial intelligence and machine learning facilitates accurate prediction of breeding values, identification of causal genes, biomarker discovery, and implementation of precision livestock breeding strategies. Such approaches are expected to accelerate genetic improvement while enhancing productivity, resilience, and sustainability.

**Figure 2:**
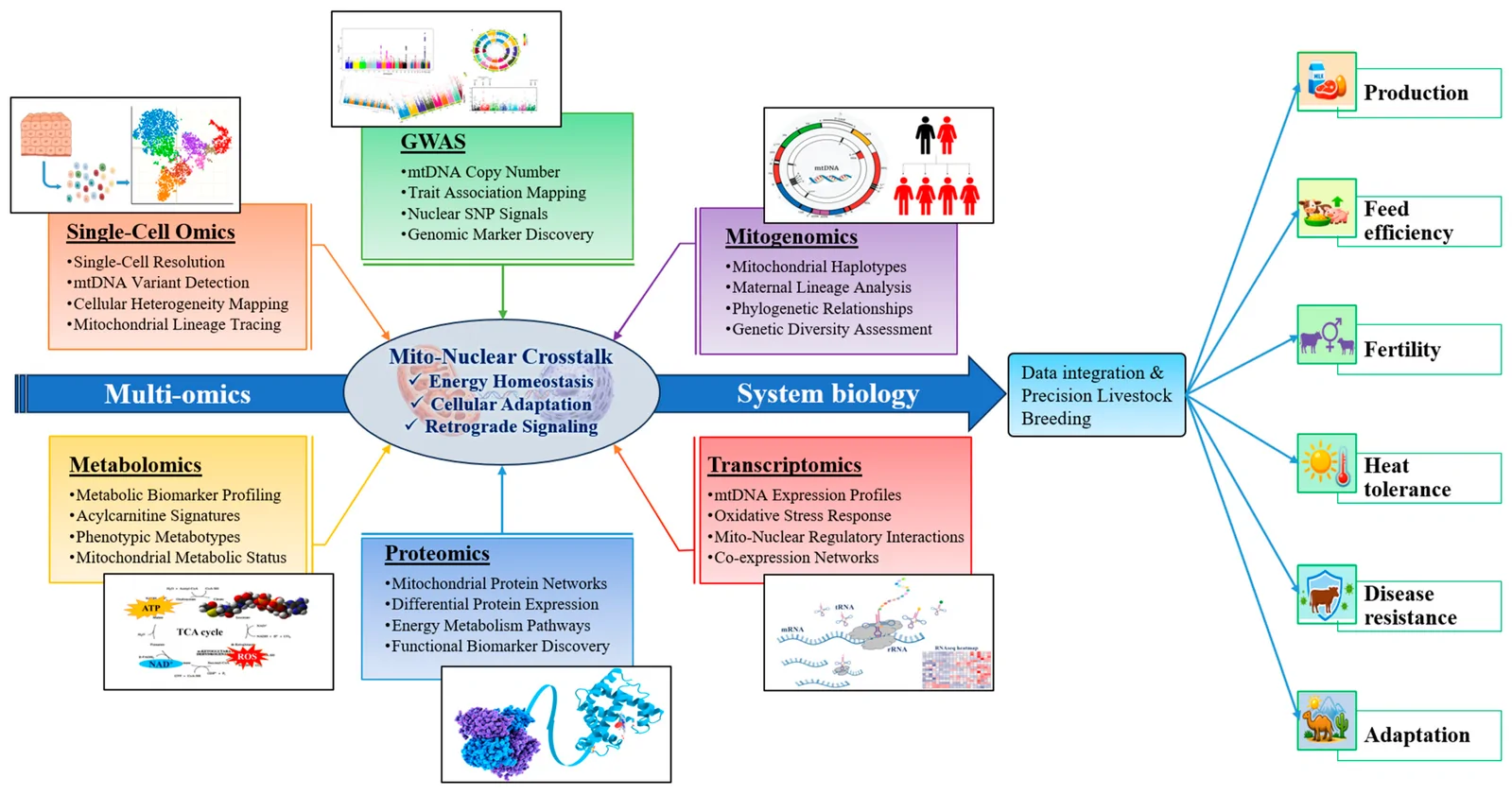
Conceptual framework illustrating the integration of mitogenomics with multi-omics data to understand mito–nuclear interactions and support precision livestock breeding.

Figure 2 presents a conceptual framework for integrating mitogenomic and multi-omics data to elucidate mito–nuclear interactions and facilitate their application in precision livestock breeding. The principal livestock studies employing genomic and multi-omics approaches to investigate mitochondrial variation, mito–nuclear regulation, and associated production and adaptation traits are summarized in Table 2.

As illustrated in Figure 2 and Table 2, several mtDNA-based association studies, such as those linking mtDNA copy number and haplotypes to backfat thickness in pigs, remain limited to single populations or breeds [114]. Yet, the causal relationship between mtDNA copy numbers and productive traits cannot be established because mtDNA copy numbers may be affected by cellular metabolic and physiological activities. Therefore, further validation across different breeds and populations is required before mtDNA-related markers can be widely applied in livestock breeding programs.

## 7. Precision Breeding Applications

### 7.1. Mitochondrial Markers in Animal Breeding

mtDNA markers have emerged as important tools in animal breeding because of their strict maternal inheritance, high mutation rate, and central role in cellular energy metabolism. Polymorphisms within mitochondrial regions such as the D-loop and coding genes, including cytochrome *b* (*MT-CYB*), are widely used for maternal lineage tracing, assessment of genetic diversity, pedigree verification, population structure analysis, detection of introgression, and identification of deleterious mutations in livestock populations [117]. mtDNA markers are also associated with economically important traits including milk yield, fertility, feed efficiency, growth, carcass quality, heat tolerance, reproductive performance, and metabolic efficiency because mitochondria regulate OXPHOS, ATP production, apoptosis, and stress-response pathways [94,118]. Furthermore, mtDNA is transmitted maternally (from the dam); therefore, it is important to consider the influence of maternal lineage in livestock. Maternal haplotypes may regulate adaptation, metabolic efficiency, fertility, and production traits independently of the nuclear genome. Hence, including maternal information during the genetic evaluation process can improve the accuracy of the selection process for maternally inherited and metabolically important traits [9,119].

Recent advances in mitochondrial genomics have expanded the application of mtDNA markers in precision livestock breeding. Studies have shown that mtDNA haplotypes and copy number variation influence bioenergetic efficiency, metabolic adaptation, and productive performance, supporting their integration into breeding and genomic selection programs [120,121,122]. Incorporating mitochondrial SNPs into commercial SNP chips has enabled routine inclusion of mitogenome information in cattle breeding and conservation genetics [123]. Fortuna et al. (2024) demonstrated that mtDNA variation may explain up to 5% of phenotypic variation in milk production traits and that inclusion of mitochondrial information improved the accuracy of estimated breeding values and genetic gain [9]. Similarly, Gibson et al. (1997) emphasized that failure to account for cytoplasmic inheritance may bias genetic evaluations [119]. Functional studies further support the relevance of mtDNA variation in livestock improvement. Kinoshita et al. (2018) demonstrated that specific mtDNA haplotypes in Holstein cattle were associated with metabolic adaptation during early lactation [30], whereas Mezzadra et al. (2005) reported negligible effects of mtDNA variation on preweaning growth traits in Hereford cattle [124], suggesting that mitochondrial effects may vary depending on breed and trait under investigation. These differences could be attributed to the physiological traits being considered, since lactation requires considerably higher energy metabolism by mitochondria compared with preweaning growth. Also, possible differences in breed background and the effect on mito–nuclear interactions could influence these contrasting findings. These findings necessitate more research across multiple breeds and larger sample sizes to determine the influence of mtDNA variants on important economic traits.

### 7.2. Genomic Selection and Personalized Breeding Incorporating mtDNA

Integration of mtDNA into genomic selection has emerged as an advanced strategy for improving genetic evaluation by incorporating both nuclear and mitochondrial genomic information. Conventional genomic selection primarily relies on nuclear SNP markers; however, mtDNA contributes significantly to OXPHOS, ATP production, metabolic adaptation, and stress response, which directly influence economically important traits such as milk yield, fertility, feed efficiency, heat tolerance, and reproductive performance. Studies have shown that mitochondrial haplotypes and mtDNA copy number variations are associated with productive and adaptive traits in livestock, supporting their inclusion in genomic prediction models [120,121,122]. Fortuna et al. (2024) demonstrated that incorporation of mitochondrial genomic information into dairy cattle genomic evaluations improved the accuracy of estimated breeding values and enhanced genetic gain, particularly for females and metabolically demanding traits [9]. Similarly, Schutz et al. (1994) reported significant associations between mtDNA polymorphisms and milk yield, milk fat production, reproductive efficiency, and health traits [125]. Because mtDNA is maternally inherited without recombination, incorporation of mitochondrial markers enables more precise evaluation of maternal lineages, cytoplasmic inheritance, metabolic efficiency, and mito–nuclear compatibility, thereby supporting personalized breeding strategies aimed at improving productivity, resilience, and long-term sustainability. An important question is whether mitochondrial effects act additively alongside nuclear breeding values or mainly through interactions with the nuclear genome. Current genomic evaluation models (e.g., GBLUP) generally consider nuclear genomic information, while mitochondrial effects are often ignored or modeled separately. However, studies indicate that favorable combinations of mitochondrial and nuclear genomes may also influence economically important traits [54,55]. Fortuna et al. (2024) used simulations to model the breeding value components of nuclear DNA (nDNA) and mitochondrial DNA (mtDNA). They demonstrated that females should be selected based on both nDNA and mtDNA, whereas males should only be selected based on nDNA [9]. However, their model considered the two components as separate breeding value components and did not evaluate genotype-by-mitotype interactions, which are largely unexplored in livestock. Moreover, incorporating mtDNA into genomic prediction remains challenging, as mtDNA is maternally inherited without recombination and represents only a few haplotypes in most livestock populations, thus making estimation of mitochondrial effects more difficult. Brajkovic et al. (2025) demonstrated that the mitogenome explained 5 to 7% of the phenotypic variation in milk production traits in Croatian Holsteins [10]. They indicated that the maternal lineages used in the cytoplasmic model did not adequately represent the variation in the cattle mitogenome, since there could be identical or phylogenetically related maternal lineages. Thus, they emphasized the importance of employing larger populations with complete mitogenome sequences to confirm these results before mitochondrial information can be used in livestock breeding programs routinely.

### 7.3. Climate-Smart Livestock Breeding and Marker-Assisted Selection

mtDNA plays an important role in climate-smart livestock breeding because mitochondria regulate OXPHOS, thermogenesis, ATP production, and stress-response pathways that determine adaptability to climatic stressors such as heat, drought, nutritional scarcity, and disease challenge. Variations in mtDNA haplotypes influence mitochondrial efficiency, ROS balance, and metabolic resilience, thereby affecting adaptive traits including heat tolerance, feed efficiency, fertility, immune response, and productivity under stressful environments. Passamonti et al. (2021) emphasized that genes involved in energy metabolism and physiological adaptation are critical targets for genomic and MAS programs aimed at improving livestock resilience to climate change [126]. Similarly, Scerri et al. (2023) highlighted the potential role of mitochondrial genotypes in improving cattle heat tolerance [127], while Freitas (2022) stressed that genetic improvement for climatic resilience and metabolic efficiency is essential for sustainable livestock production under global warming scenarios [128]. Integrating mtDNA markers with nDNA markers can substantially enhance the efficiency and accuracy of MAS by enabling simultaneous evaluation of nuclear and cytoplasmic genetic contributions to economically important traits.

Nuclear genes encode the majority of mitochondrial proteins, whereas mtDNA encodes essential components of the respiratory chain; therefore, favorable mito–nuclear interactions are critical for efficient cellular metabolism and physiological performance. Kinoshita et al. (2018) demonstrated that mitochondrial haplotypes influence metabolic adaptation during early lactation [30], whereas Gibson et al. (1997) emphasized that failure to account for cytoplasmic inheritance may reduce the accuracy of genetic evaluations [119]. Integration of mitochondrial and nuclear markers can therefore improve selection for maternal, adaptive, and metabolically demanding traits, particularly in breeding programs targeting climate resilience, reproductive efficiency, feed conversion efficiency, and long-term productivity. Furthermore, mito–nuclear compatibility is an important parameter that should be considered during crossbreeding programs because the mtDNA inherited from the mother may not be optimally matched with the biparentally inherited nuclear genome. This type of incompatibility can induce fertility-related problems and disturb energy metabolism, adaptation, and productivity. Hence, to improve the outcomes from a crossbreeding program, information on maternal lineages can complement nuclear genomic selection [43,44,92].

### 7.4. Precision Livestock Farming

Integration of mtDNA information can significantly enhance PLF by improving the monitoring and prediction of animal performance, health, metabolic efficiency, and environmental adaptability. PLF integrates sensor technologies, automated systems, artificial intelligence, and genomic tools to optimize productivity and animal welfare. Since mitochondria regulate OXPHOS, ATP production, thermoregulation, and stress-response pathways, mtDNA variation directly influences traits such as feed efficiency, fertility, milk production, heat tolerance, immune response, metabolic resilience, and disease susceptibility. Integration of mtDNA information with genomic selection, metabolomics, transcriptomics, and sensor-based phenotyping can improve the identification of animals with superior metabolic efficiency and adaptive capacity. Studies by Kinoshita et al. (2018) and Fortuna et al. (2024) demonstrated that mitochondrial variation influences metabolic adaptation and improves prediction accuracy for metabolically demanding traits in dairy cattle [9,30]. In practical PLF applications, mtDNA information can support individualized animal management by optimizing breeding strategies, early prediction of metabolic stress, enabling precision nutritional interventions, and identifying climate-resilient maternal lineages. Consequently, incorporating mtDNA information into PLF frameworks can improve animal welfare, production efficiency, climate adaptability, and sustainability of livestock production systems.

## 8. Challenges and Limitations

Functional coordination between the mitochondrial (mtDNA) and nuclear genomes (nDNA) is mainly controlled by mito–nuclear interactions in livestock [129]. Cellular metabolism, energy production, immunity, fertility, and environmental adaptation are controlled by mitochondrial activity [130,131]. However, the characterization and practical application of mito–nuclear interactions are constrained by biological complexities, including dual-genome coordination, maternal inheritance, heteroplasmy, mito–nuclear incompatibility, and environmental sensitivity, as well as by limited reference populations, difficulties in functional validation, and computational limitations [55]. The biological basis, consequences, and research priorities associated with these challenges are summarized in Table 3. Although there have been an increasing number of studies exploring the interaction between mitochondrial and nuclear genes in livestock, much of the current evidence is derived from association studies that were conducted within a single breed or population. In addition, the maternal inheritance of mtDNA and relatively small reference populations can make it challenging to distinguish true mitochondrial effects from confounded maternal/common-environment effects.

### 8.1. Complexity of Dual Organelles Genome Interactions

Mitochondrial functions controlled by two distinct organelle genomes faced major challenges. This may be because mtDNA encodes only a few mitochondrial proteins, and they rely on nuclear genes to encode most mitochondrial proteins. Therefore, any slight incompatibilities in this coordination can alter ATP production, increase oxidative stress, and impair the health of the animal.

### 8.2. Challenges Due to Maternal Inheritance of mtDNA

Unlike nuclear genes, mtDNA is primarily inherited from the mother in most animals. This creates several difficulties, as it reduces selection pressure [7] and means a superior male cannot transmit its mitochondrial genome to progeny [58]. Productivity evaluation may be confounded by matrilineal effects of mtDNA [10]. This limits understanding of genomic selection models primarily based on the biparental nuclear inheritance [132]. Furthermore, the maternal inheritance of mtDNA contributes to several practical challenges for its utilization in conventional livestock breeding programs [7,132]. Because mtDNA is mainly transferred to offspring from the maternal lineage [19,20,24], it shows less or no recombination and no paternal inheritance under normal conditions [20,133]. However, mitochondrial genetic variation cannot be maintained by conventional mating strategies [132]. This may limit the response to genomic selection. Conventional mating strategies mostly depend on nuclear genome-based estimated breeding values (EBVs) and genomic estimated breeding values (GEBVs). It becomes difficult to assess the independent contribution of mtDNA due to nuclear genetic effects [9,119]. Additionally, OXPHOS and cellular energy metabolism data are rarely considered in conventional mating programs [9,123]. Current genomic evaluation pipelines and SNP-based prediction models, especially those focused on nuclear markers, are underutilized due to the lack of proper utilization of mitochondrial genetic information in breeding decisions [120,121,122].

### 8.3. Mito–Nuclear Discordance or Incompatibility During Crossbreeding

Hybrid offspring resulting from hybridization between various races and subspecies may inherit mitochondrial genomes that are unable to interact with the nuclear genomes of such hybrid progeny. This mismatch in interactions between nuclear and mitochondrial gene combinations can lead to “hybrid breakdown”. Studies suggest that the hybrid breakdown may lead to reduced fertility, lower feed efficiency, metabolic defects, impaired thermotolerance, reduced disease resistance, or developmental failure [92].

### 8.4. Challenges with a Higher Rate of mtDNA Mutation

mtDNA generally exhibits mutation rates several-fold higher than nDNA [134]. One possible reason is high levels of mitochondrial ROS and decreased DNA repair mechanisms. This leads to substantial accumulation of variants, heteroplasmy (presence of multiple mtDNA variants), instability across generations, and unpredictable phenotypic expression [25,135].

### 8.5. Difficulty in Identifying Functional Differences

The impact of mtDNA polymorphisms seems to be neutral because there is no efficient repair mechanism, and the mitochondrial genome mutates at a much higher rate than the nuclear genome [136]. Purifying selection helps maintain the neutral nature of polymorphisms by eliminating less effective mutations. Hence, many mitochondrial polymorphisms are considered selectively neutral, although some may influence adaptive traits under specific environmental conditions. Although many polymorphisms are neutral, some may influence metabolism, milk production, fertility, and adaptation to heat or altitude. Therefore, it is indeed difficult to distinguish between functional mutations and neutral variations within mtDNA.

### 8.6. Availability of Small Reference Populations and Data Scarcity

Compared to conventional nuclear genomic databases, the mitochondrial datasets are limited in livestock [11]. This leads to major challenges, including insufficient sequencing of maternal lineages, a lack of standardized mtDNA reference panels, inadequate phenotype-genotype integration, and underrepresentation of indigenous breeds. These limitations reduce the statistical power for identifying reliable mito–nuclear markers, which may affect economically important traits.

### 8.7. Sensitivity of Mito–Nuclear Interactions to Environmental Stress

The crosstalk between the mitochondria and nuclear genome is highly dependent on environmental factors such as nutrition, temperature, disease pressure, altitude, and salt management systems [137,138,139]. For example, crosstalk between the mitochondrial and nuclear genome is advantageous under tropical heat stress conditions but may not be beneficial under temperate climatic conditions. Therefore, a breeding strategy developed for one environmental condition may not be useful for another condition.

### 8.8. Relationship Between Productivity and Adaptation

Directional selection, driven by high intensity for higher production, can reduce genetic diversity in the mitochondrial genome of livestock populations. This type of selection mainly focuses on improving production traits and generates high-yielding livestock [140]. Consequently, the adaptive capacity of livestock may be compromised.

### 8.9. Computational and In Silico Limitations

Crosstalk studies on mito–nuclear interactions generate large, complex datasets [129]. To manage these datasets, various advanced analytical approaches, such as machine learning, systems biology, network genomics, and high-performance computational infrastructure, are used [130]. Hence, implementing such technologies in livestock breeding programs remains challenging, especially in developing countries, because of limited computational resources, insufficient availability of high-throughput sequencing facilities, and a shortage of skilled bioinformaticians. These limitations hinder the effective integration of mito–nuclear crosstalk data into livestock improvement programs. Collectively, these challenges demonstrate that the application of mitochondrial information in livestock breeding is constrained not by a single limitation, but by interconnected biological, statistical, and implementation barriers. Progress will require larger and more diverse reference populations, accurate quantification of heteroplasmy, joint modeling of mitochondrial and nuclear effects, functional validation of candidate variants, and evaluation across contrasting production environments. Standardized multi-omics and computational pipelines will also be essential for translating mito–nuclear research into reliable genomic prediction and precision-breeding applications.

## 9. Future Perspectives

The introduction of precision livestock farming (PLF) has made the selection of superior animals more efficient. It involves real-time observation of individual animals through sensors, IoT (Internet of Things) devices, and big data analytics, providing ongoing, comprehensive insights into animal health, behavior, and productivity [141]. Analytical processes are applied to the dataset to identify the specific genetic markers associated with the required phenotypes. This information could help enhance breeding programs, as animals that display the most suitable genotypes for these traits could be selected. It provides a broad picture of the genome, which helps identify genetic variation associated with complex traits that can be simple or complex. It offers an improved ability to select breeding animals to breed, since WGS reveals all relevant genetic variations, compared with those that are already documented [142]. Future genomic prediction models are expected to incorporate whole-genome variants, mitochondrial haplotypes, and multi-omics biomarkers to improve prediction accuracy for complex traits. Such integrated prediction models will enhance the selection of superior breeding animals by capturing both additive genetic effects and functional biological variation. Achieving this vision depends on a computational framework capable of managing data at this scale and complexity, where artificial intelligence (AI) becomes essential. Machine learning (ML) algorithms have been applied to genomic prediction in dairy cattle, beef cattle, pigs, and poultry, valued for their ability to capture non-additive genetic effects and complex marker interactions that traditional models often miss. However, ML models do not yet consistently outperform conventional methods like GBLUP, and their broader adoption in animal breeding remains constrained because of data limitations, interpretability challenges, and a lack of standardization [143]. Nevertheless, AI and ML are expected to increasingly integrate genomic, transcriptomic, proteomic, metabolomic, phenomic, and environmental datasets. Combined with PLF technologies, such as wearable sensors, computer vision, automated monitoring systems, and IoT-based devices, AI will enable real-time health monitoring, disease prediction, behavioral assessment, and precision management.

Building on this integration, digital twins, the virtual models of individual animals that continuously update with genomic, physiological, environmental, and production data, will enable simulation of management strategies and prediction of animal performance before real-world application [144]. Unlike static models, digital twins are dynamic, constantly refreshed with new data, serving as dynamic representations rather than fixed representations [145]. As AI-driven precision livestock farming (PLF) systems advance, digital twins are set to become the key interface through which breeders and farm managers simultaneously utilize genomic and real-time phenotypic information. A critical application of this integrated framework is breeding for climate resilience. Increasing global temperatures are expected to exacerbate heat stress risks in most livestock production systems, disproportionately affecting extensive and smallholder operations in tropical and lower-latitude regions, where adaptation costs are greatest [146]. Genomic methodologies, such as genome-wide association studies and selection-signature analyses, have identified candidate genes and haplotypes associated with thermotolerance. Indigenous or locally adapted breeds often harbor unique alleles shaped by prolonged exposure to heat stress, representing a valuable genetic resource for breeding. Mitochondrial haplotypes are particularly pertinent because mitochondrial OXPHOS is temperature-sensitive. For instance, studies in Holstein dairy cattle have correlated specific mtDNA haplotypes with variation in liver fat metabolism and metabolic adaptive capacity [30], and broader genomic analyses have highlighted mtDNA maintenance as a key pathway under selection for thermal tolerance in cattle. This exemplifies a polygenic, environmentally influenced trait that benefits from AI-enhanced genomic prediction combined with PLF-enabled real-time phenotyping, as neither genomics nor sensor data alone can comprehensively capture the expression of genetic resilience under fluctuating environmental conditions. Collectively, the integration of artificial intelligence, precision livestock farming, digital twins, whole-genome sequencing, mitochondrial haplotype analysis, and multi-omics genomic prediction constitutes the forefront of animal breeding innovation. These technologies are increasingly interdependent rather than isolated; AI provides the computational capacity to integrate genomic and phenotypic data streams, PLF and digital twins offer continuous, individual-level data for model input, and genomic and mitochondrial markers serve as biological targets for selection. Their synergistic application holds promise to accelerate the breeding of animals that are not only more productive but also genetically equipped to endure the challenges posed by a changing climate.

Summary Box

1. Mito–nuclear interactions are crucial for mitochondrial function, energy metabolism, and cellular homeostasis.
2. Mito–nuclear compatibility affects fertility, Disease Resistance, Adaptation and Animal Productivity.
3. The use of multi-omics technologies has improved our knowledge about mito–nuclear regulation and its contribution to economically important traits.
4. Precision breeding, climate resilience, and sustainable livestock production can all be enhanced by incorporating mitochondrial genomic information into genomic selection.

## 10. Mito–Nuclear Interactions in Non-Livestock Species

*Drosophila subobscura* provides a valuable model for investigating the maintenance of mitochondrial DNA (mtDNA) variation and the adaptive significance of mito–nuclear interactions. Experimental mito–nuclear lines generated through backcrossing enable the assessment of fitness effects and balancing selection mechanisms, offering important insights into the maintenance of mtDNA diversity in natural populations [147]. McDiarmid et al. (2024) [148] demonstrated that mito–nuclear interactions can have significant fitness consequences even at low mtDNA divergence (~1%), potentially driving asymmetric mitochondrial introgression in hybrid populations. These findings suggest that mito–nuclear incompatibilities may be widespread across taxa and play an important role in adaptation and hybridization. Future studies should identify the underlying genes and molecular pathways and investigate their effects across developmental stages to better understand the evolutionary significance of mito–nuclear interactions [148]. Identifying the genes underlying mito–nuclear interactions will facilitate the elucidation of their molecular pathways and evolutionary mechanisms, providing valuable insights into the evolution of mito–nuclear coadaptation [149,150,151]. The sensitivity of mitochondrial function to genetic variation, its central role in cellular energy production, and its conservation across diverse taxa suggest that mito–nuclear interactions are a major evolutionary force influencing adaptation and hybridization [38].

## 11. Conclusions

Mito–nuclear interactions constitute a fundamental axis of biological regulation in livestock, governing the coordinated function of two physically separate genomes to sustain OXPHOS, energy metabolism, and cellular homeostasis. This review has shown that compatibility between mitochondrial and nuclear genomes shapes a wide spectrum of economically important traits—including growth, milk yield, feed efficiency, fertility, disease resistance, and adaptation to heat, altitude, and other environmental stressors—through mechanisms such as co-evolution, anterograde and retrograde signaling, ROS-mediated crosstalk, epigenetic regulation, and the balance between mitophagy and biogenesis. Advances in genomics and multi-omics—spanning mitogenomics, transcriptomics, proteomics, metabolomics, and single-cell technologies—have begun to reveal how mito–nuclear variation translates into phenotypic outcomes, yet the field remains constrained by small and underrepresented reference populations, unresolved maternal-lineage effects, tissue-specific heteroplasmy, and a scarcity of functionally validated variants. Overcoming these constraints will require larger, multi-breed datasets, standardized analytical pipelines, and deliberate modeling of mito–nuclear (rather than purely additive nuclear) effects within genomic evaluation frameworks. Doing so would allow mitochondrial information to be routinely incorporated into genomic selection, crossbreeding design, and precision livestock farming, complementing AI-driven and multi-omics-enabled breeding strategies. As these tools mature, explicitly accounting for mito–nuclear compatibility offers a tangible path toward livestock that are simultaneously more productive, fertile, disease-resistant, and resilient to a changing climate.

## Figures and Tables

**Table 1:** Biological mechanisms and breeding implications of mito–nuclear interactions associated with livestock adaptation and productivity.

Trait/Area	Key Findings	Species	Practical Implication	Reference
Heat adaptation	Mitochondrial biogenesis; enhanced OXPHOS	Dairy cattle	Selection for Thermotolerance	[27,41]
High-altitude adaptation	Hypoxia tolerance through OXPHOS gene adaptations	Bar-headed goose	Selection for high-altitude and hypoxic conditions	[42]
Environmental adaptation	Mito–nuclear incompatibility reduces metabolic efficiency, fecundity, and lifespan	Multiple species	Improve adaptation and crossbreeding strategies	[43,44]
Disease resistance	Disruption of mito–nuclear interactions contributes to neurodegenerative disorders	Multiple species	Genetic screening and disease risk assessment	[28]
Immunity and metabolism	TCA cycle metabolites regulate immune response and thermogenesis	Multiple species	Biomarkers for immune competence and metabolic resilience	[45]
Mitochondrial disease	*ND4L* mutation (*T10663C*) associated with exophthalmia	Cattle	Detection of pathogenic mtDNA variants in breeding populations	[46]
Neuropathy	Mitochondrial dysfunction induced by hypoglycin-A causes atypical neuropathy	Horse	Early diagnosis and prevention of mitochondrial disorders	[47]
Metabolic disorders	Oxidative stress and mitochondrial dysfunction linked with inflammatory and metabolic disorders	Hens and pigs	Selection for stress resilience	[48]
Milk yield and composition	Mitogenome explains 5–7% of phenotypic variation	Holstein cattle	Improve genomic prediction	[9]
Lactation performance	Mitochondrial enzyme activity associated with parity and lactation performance	Holstein cattle	Selection for improved lactation performance	[49]
Growth and feed efficiency	Mitochondrial bioenergetics influences growth, muscle development, and feed efficiency	Livestock species	Selection for improved feed efficiency and average daily gain	[29]
Aging and health	Accelerated epigenetic aging associated with disease susceptibility and methylation changes in *MAB21L1*	Cattle	Biomarkers for longevity prediction	[50]

**Table 2:** Summary of the principal genomic and multi-omics studies investigating mitochondrial and mito–nuclear functions in livestock.

Study/Approach	Livestock Species (Sample Size)	Mitochondrial Marker/Omics	Trait Investigated	Key Quantitative Findings	Limitations	Reference
GWAS + mtDNA copy number	Pigs (*n* = 234)	mtDNA copy number, GWAS	Carcass traits	Higher mtDNA copy number was associated with increased backfat thickness and muscle pH, whereas loin thickness and lean percentage decreased.	Single population; validation required	[113]
mtDNA association	Pigs (*n* = 234)	mtDNA mutations, haplotypes	Backfat thickness	Maternal haplotypes significantly associated with carcass fat deposition.	Limited validation across breeds	[114]
Population mitogenomics	Cattle (*n* = 1883; 156 breeds)	Complete mitogenome	Maternal diversity	Identified 11 haplogroups and 1309 haplotypes, demonstrating extensive mitochondrial diversity.	Functional validation lacking	[115]
Multi-tissue transcriptomics	Cattle (441 animals; 91 tissues)	RNA-seq	Mitochondrial gene regulation	Identified mitochondrial network containing 13 protein-coding genes and 15 tRNAs.	Functional studies needed	[112]
Integrated transcriptomics	Chinese Simmental cattle (*n* = 6)	RNA-seq + WGCNA	Fatty acid metabolism	2 co-expression modules and 38 candidate genes identified.	Small sample size	[116]
Comparative proteomics	Yak, dzo, cattle (192, 191, and 281 proteins in the yak-dzo, yak-cattle, and dzo-cattle groups)	Mitochondrial proteomics	Hypoxia adaptation	376 differentially expressed proteins identified.	Bioinformatic validation only	[105]
Feed efficiency	Cattle (cattle and dzo-cattle groups)	Gene expression	Residual feed intake	244 DEGs, including 38 mitochondrial genes enriched in OXPHOS.	Needs larger validation populations	[106]
Metabolomics	Dairy cattle	Metabolite profiling	Productive lifespan	Long-chain acylcarnitines and biogenic amines predicted productive lifespan before lactation.	Mainly dairy cattle	[109]
Single-cell omics	Livestock (limited)	scMito-seq	Heteroplasmy	Detects rare mtDNA variants at single-cell resolution.	Amplification bias and incomplete genome coverage	[111]
Network biology	Beef cattle	Co-expression network	Feed efficiency	Identified PPARA as a major mitochondrial regulator.	Requires experimental validation	[112]

**Table 3:** Major challenges and limitations in studying and applying mito–nuclear crosstalk in livestock.

Challenge	Biological or Methodological Basis	Major Consequence for Livestock Research and Breeding	Priority for Resolution
Dual-genome coordination	Mitochondrial function depends on coordinated expression of mtDNA- and nDNA-encoded proteins, particularly within OXPHOS complexes.	Even minor incompatibilities may impair ATP production, increase oxidative stress, and reduce physiological performance.	Joint analysis of mitochondrial and nuclear variants, gene expression, and metabolic phenotypes.
Maternal inheritance of mtDNA	mtDNA is predominantly maternally inherited, has little or no recombination, and cannot normally be transmitted through superior sires.	Conventional nuclear-genome-based EBVs and GEBVs may not adequately capture mitochondrial or maternal-line effects.	Incorporation of maternal lineage, mitochondrial haplotypes, and mito–nuclear interaction terms into genetic evaluations.
Mito–nuclear incompatibility during crossbreeding	Crossbreeding may combine a mitochondrial haplotype with a nuclear genetic background with which it has not co-evolved.	Incompatible combinations may reduce fertility, feed efficiency, thermotolerance, disease resistance, or developmental viability.	Evaluation of reciprocal crosses and specific mitochondrial-haplotype × nuclear-genotype combinations before widespread use.
High mtDNA mutation rate and heteroplasmy	mtDNA has a relatively high mutation rate, and different mitochondrial variants may coexist within an animal or tissue.	Variant frequencies and phenotypic effects may differ among tissues, individuals, and generations, complicating genetic evaluation.	Deep sequencing, tissue-specific sampling, heteroplasmy quantification, and standardized detection thresholds.
Difficulty distinguishing functional from neutral variants	Many mtDNA polymorphisms are selectively neutral or occur in linkage with causal variants.	Statistical associations may not represent causal effects on production, reproduction, health, or adaptation.	Functional validation using transcriptomic, proteomic, metabolomic, cellular, and experimental evidence.
Small reference populations and data scarcity	Livestock mitogenomic datasets contain relatively few animals, maternal lineages, indigenous breeds, and matched phenotypes.	Low statistical power, population-specific estimates, and poor reproducibility limit marker discovery and genomic prediction.	Development of large, multi-breed reference populations with standardized phenotypes and complete mtDNA information.
Environmental sensitivity	Mito–nuclear effects may vary with temperature, nutrition, altitude, disease pressure, and management conditions.	Associations detected in one production environment may not be transferable to another environment.	Multi-environment studies and explicit modeling of mito–nuclear × environment interactions.
Productivity–adaptation trade-off	Intense selection for production may reduce mitochondrial diversity or favor genotypes that perform poorly under environmental stress.	Short-term production gains may be accompanied by reduced resilience, fertility, or adaptive capacity.	Multi-trait selection balances productivity, fitness, mitochondrial diversity, and environmental resilience.
Computational and implementation limitations	Mito–nuclear research requires integration of genomic, transcriptomic, proteomic, metabolomic, environmental, and phenotypic datasets.	Complex analyses, limited infrastructure, poor data standardization, and a shortage of trained personnel hinder practical application.	Standardized analytical pipelines, interoperable databases, accessible computational tools, and capacity building.

OXPHOS, oxidative phosphorylation; mtDNA, mitochondrial DNA; nDNA, nuclear DNA; EBV, estimated breeding value; GEBV, genomic estimated breeding value.

## Data Availability

No new datasets were generated or analyzed during the current study. Data sharing does not apply to this article.

## Abbreviations

mtDNA	mitochondrial DNA
nDNA	*nuclear DNA*
OXPHOS	oxidative phosphorylation
ROS	reactive oxygen species
GWAS	Genome-wide association studies
PLF	Precision livestock farming
PPARγ	peroxisome proliferator-activated receptor gamma
PGC-1α	Peroxisome proliferator-activated receptor gamma coactivator 1-alpha
NRF	Nuclear respiratory factors
ETC	Electron transport chain
MAPK	Mitogen-activated protein kinase
NF-κB	Nuclear factor kappa B
HIF-1α	Hypoxia inducible factor 1α
SAM	S-adenosylmethionine
PINK1	Phosphatase and tensin homolog (PTEN) induced kinase 1
MAP	microtubule-associated protein 1
LC3	light chain 3
LIR	light chain 3 interacting regions
ERRα	Estrogen-related receptor alpha
PPARα	peroxisome proliferator-activated receptor alpha
TFAM	Mitochondrial transcription factor A
LHON	Leber’s Hereditary Optic Neuropathy
NGS	Next-generation sequencing
NUMTs	Nuclear mitochondrial DNA segments
SNP	Single nucleotide polymorphism
RFI	Residual feed intake
RCA	Rolling circle amplification
WGS	Whole Genome Sequencing
GBLUP	Genomic Best Linear Unbiased Prediction
